# Reconstitution of human pyroptotic cell death in *Saccharomyces cerevisiae*

**DOI:** 10.1038/s41598-023-29464-5

**Published:** 2023-02-22

**Authors:** Yanhao Ji, Christine J. Hawkins

**Affiliations:** Department of Biochemistry and Chemistry, La Trobe Institute for Molecular Science, La Trobe University, Bundoora, VIC Australia

**Keywords:** Cancer, Cell biology

## Abstract

Pyroptosis is a lytic form of programmed cell death induced by the activation of gasdermins. The precise mechanism of gasdermin activation by upstream proteases remains incompletely understood. Here, we reconstituted human pyroptotic cell death in yeast by inducible expression of caspases and gasdermins. Functional interactions were reflected by the detection of cleaved gasdermin-D (GSDMD) and gasdermin-E (GSDME), plasma membrane permeabilization, and reduced growth and proliferative potential. Following overexpression of human caspases-1, -4, -5, and -8, GSDMD was cleaved. Similarly, active caspase-3 induced proteolytic cleavage of co-expressed GSDME. Caspase-mediated cleavage of GSDMD or GSDME liberated the ~ 30 kDa cytotoxic N-terminal fragments of these proteins, permeabilized the plasma membrane and compromised yeast growth and proliferation potential. Interestingly, the observation of yeast lethality mediated by co-expression of caspases-1 or -2 with GSDME signified functional cooperation between these proteins in yeast. The small molecule pan-caspase inhibitor Q-VD-OPh reduced caspase-mediated yeast toxicity, allowing us to expand the utility of this yeast model to investigate the activation of gasdermins by caspases that would otherwise be highly lethal to yeast. These yeast biological models provide handy platforms to study pyroptotic cell death and to screen for and characterize potential necroptotic inhibitors.

## Introduction

Pyroptosis is a form of programmed cell death characterized by plasma-membrane rupture and release of proinflammatory contents, which has been observed in multiple microbial infections and diseases^1–4^. Pyroptosis can be triggered by innate immune responses through distinct pathways^5^. Gasdermins (GSDMs) are a superfamily of pore-forming executioner proteins that typically consist of a cytotoxic N-terminal domain (GSDM-N) and an auto-inhibitory C-terminal domain (GSDM-C) connected by a linker structure, except for GSDMF (PJVK/DFNB59), in which the C-terminal domain is replaced by a zinc-finger^6,7^. GSDMF has not been demonstrated to possess cell-killing activity. The other full-length gasdermins remain inactive in the cytosol until a C-terminal inhibitory fragment is proteolytically removed, activating the lytic potential of their N-terminal domains^8^. Pyroptosis was first identified as an inflammatory type of cell death, mediated by GSDMD, to promote pathogen clearance^9,10^. The most well-studied form of pyroptosis, which is triggered by activation of gasdermin D (GSDMD), can be mediated by inflammatory caspases, via two pathways^9,10^. The canonical pathway occurs in response to the recognition of microbial infections or danger signals by intracellular pattern-recognition receptors (PRRs), such as Nod-Like Receptors (NLRs) or Absent In Melanoma (AIM)-like receptors (ALRs), which initiate the assembly of inflammasomes in the cytosol, where caspase-1 is recruited and activated^11,12^. The noncanonical pathway occurs following LPS-induced direct activation of caspases-4 or -5 through their caspase activation and recruitment domains (CARD’s)^13,14^. The activated inflammatory caspases then activate GSDMD via cleavage at Asp275 (^272^FLTD^275^), generating the ~ 30 kDa active N-terminal fragment, which leads cells to a lytic death by forming pores on the plasma membrane^10,15^.

Although pyroptosis is primarily triggered by the activation of inflammatory caspases through innate immune responses, recent studies dissecting the physiological and molecular crosstalk of programmed cell death revealed the role of apoptotic caspases in inducing pyroptosis under certain conditions. Activation of Tumor necrosis factor receptor 1 (TNFR1) via ligand binding initiates the assembly of various high-molecular-weight signaling complexes^16^. Formation of Complex I on the cytosolic side of the plasma membrane promotes prosurvival and proinflammatory gene induction via the NF-ĸB pathway^17,18^. Inhibition of protein synthesis downstream of NF-ĸB transcriptional response results in formation of Complex IIa^19,20^. Similarly, inhibition or depletion of specific Complex I effectors, such as cellular Inhibitor of Apoptosis Proteins (cIAP) 1/2, promotes the assembly of Complex IIb^21,22^. Both Complex IIa and IIb initiate autoprocessing of apoptotic initiator caspase-8, promoting apoptotic cell death^23^. Interestingly, while GSDMD is partially activated following caspase-8 activation in Complex IIa, GSDMD is largely activated by caspase-8 via direct cleavage in Complex IIb^24^. Unlike caspase-8-mediated direct cleavage of GSDMD in Complex IIa and IIb, Fas-mediated activation of caspase-8 in death-inducing signaling complex (DISC) triggers the assembly of NLRP3 inflammasome, where GSDMD is activated by caspase-1^24–26^. Furthermore, in addition to activating GSDMD, caspase-mediated cleavage at a distinct site from ^272^FLTD^275^ was shown to inactivate GSDMD^24,27^. Apoptotic caspases-3 and -7 were found to cleave GSDMD at Asp87(^84^DAMD^87^), which inactivated the cytotoxic N-terminal domain of GSDMD, and inhibited the subsequent pyroptotic cell death^24,27^.

Activation of the necroptotic effectors—Receptor-interacting serine/threonine-protein kinase 3 (RIPK3) and/or Mixed lineage kinase domain-like (MLKL)—by Toll-like receptor (TLR)- or TNFR1-induced signaling pathway, may also induce caspase-1 cleavage of GSDMD via formation of NLRP3 inflammasome^28,29^. Studies focusing on GSDME have revealed that, in cells expressing GSDME, activation of the executioner caspase-3 via either intrinsic or extrinsic apoptotic pathway can cleave and activate GSDME at Asp270 (^267^DMPD^270^), which results in pyroptosis^30,31^.

Yeast expression has long been used to study mammalian cell death proteins^32–34^. Overexpression-mediated activation of cell death effector proteins circumvents the requirements for receptor activation and signal transduction mediated by upstream adaptor proteins in mammalian cells, such that the impact on yeast viability by co-expressed recombinant proteins reflects their interactions and functions in mammalian cells^35–37^. In addition, studies of recombinant proteins in the naïve eukaryotic yeast environment enables a specific focus on the expressed proteins, avoiding crosstalk-mediated interference between various cellular pathways. For example, it has been shown that, in addition to mediating pyroptosis, caspase-8 can also mediate extrinsic apoptosis^23^, suppress necroptosis^38,39^, and regulate various non-cell death cellular pathways^40,41^.

Previously, we modeled necroptotic cell death mediated by RIPK1, RIPK3 and MLKL in yeast, demonstrating that reconstituted mammalian lytic cell death in yeast provides a tool for investigating intra- and intermolecular mechanisms of pore-forming proteins, and establishing a platform for high-throughput screening for functional domains or potential inhibitors^35^.

The aim of this study was to reconstitute pyroptotic cell death in yeast, focusing on caspase-gasdermin interactions. We demonstrate that GSDMD and GSDME acted as substrates for active caspases when heterologously co-expressed in the yeast context. We characterized the impacts of co-expressed human gasdermins and human caspases on *S. cerevisiae* viability, finding that cleaved GSDMD and GSDME were cytotoxic to yeast transformants. Collectively, this study established a heterologous yeast model for studying pyroptotic cell death, extending our understanding of the molecular basis of gasdermin-mediated pyroptosis.

## Results

### The N-terminal domains of GSDMD and GSDME induce yeast lethality

**Figure 1:**
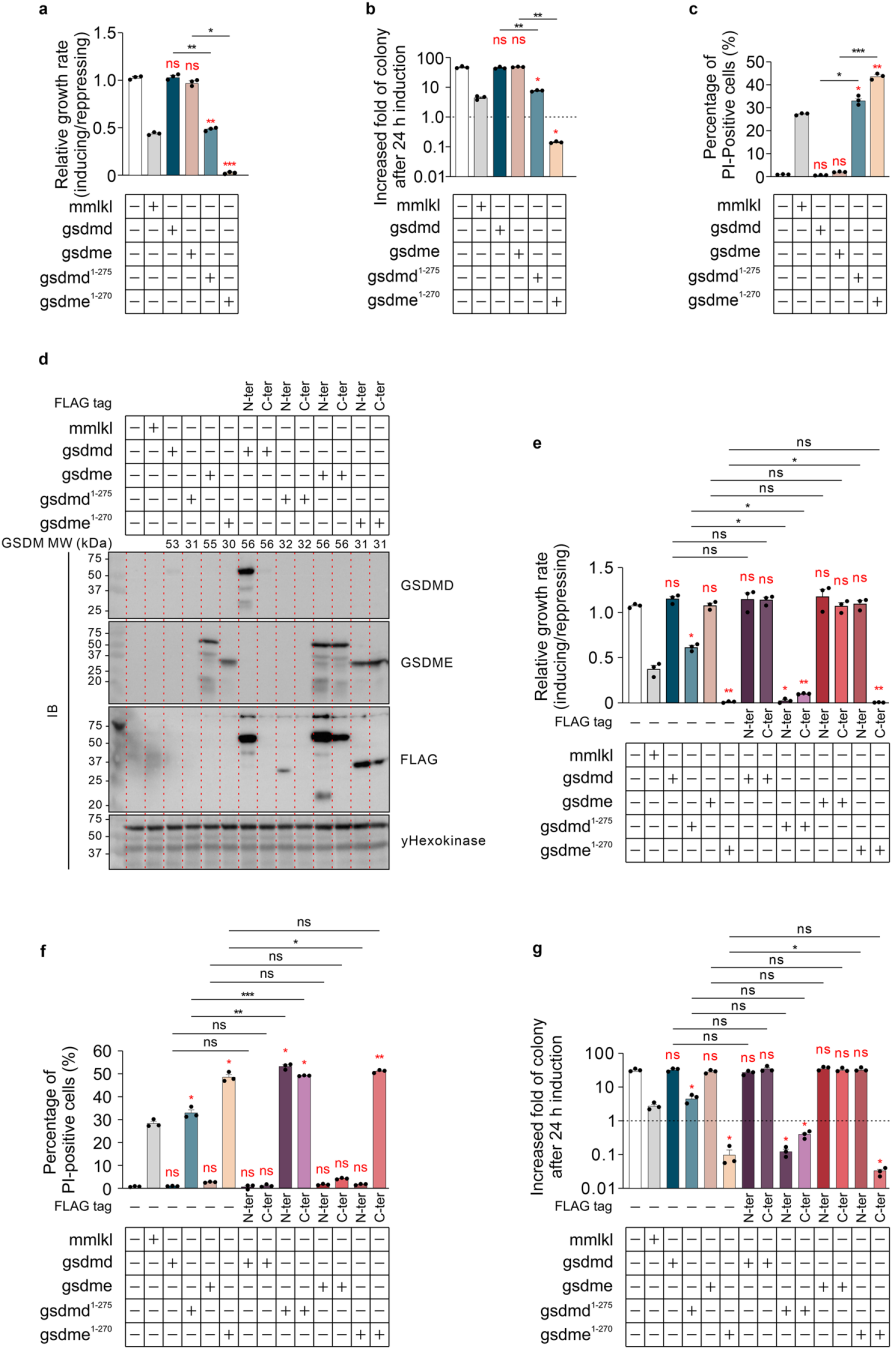
Overexpression of gasdermins in *S. cerevisiae*. Maximal growth rates of yeast bearing expression plasmids encoding (**a**) wild type or (**e**) FLAG-tagged full-length and N-terminus of GSDMD and GSDME, or empty vectors lacking transgenes, were analyzed by monitoring absorbance of yeast cultured in both inducing and repressing liquid media. Yeast transformants were grown under inducing condition for 24 h then clonogenicity and membrane integrity were assessed. Following galactose removal, yeast expressing wildtype (**b**) or FLAG-tagged (**g**) proteins were evaluated by clonogenicity assay, where yeast were diluted, and incubated on solid repressing media for 3 days before counting. The colony-forming ability of the post-induction yeast culture were assessed by increasing fold of colonies relative to each uninduced culture. The membrane integrity of yeast expressing wild type (**c**) or FLAG-tagged proteins (**f**) were analyzed by propidium iodide (PI) uptake flow cytometry. Expression of recombinant expressed wild type or FLAG-tagged GSDMD and GSDME were assessed by western blot (**d**). Predicted molecular weights of the different forms of gasdermin proteins are noted above the blots. Loading control: anti-γhexokinase. Uncropped blots are presented in Supplementary Fig. 1. Data present mean ± SEM of three independent assays. Statistical analysis of differences in growth rates, membrane integrity and clonogenic ability were compared using ANOVAs with Sidak corrections. Comparisons made between data from empty vector yeast and those expressing each transgene were labeled in red color. *, *P* < 0.05; **, *P* < 0.01; ***, *P* < 0.001; ns, not significant.

We first explored the impact of overexpressed gasdermins in *Saccharomyces cerevisiae*, investigating the toxicity of intact gasdermins or their cytotoxic N-terminal domains in the yeast context. To comprehensively determine the activities of the executors of pyroptosis in yeast, we measured the maximal growth rates, changes in membrane integrity and clonogenicity of yeast transformants overexpressing full-length and N-terminal domains of GSDMD and GSDME. Murine MLKL, which has been demonstrated to cause yeast lethality when overexpressed^35^, acted as a positive control. While neither the wild type GSDMD nor GSDME compromised yeast viability, the N-terminal truncation mutants of both gasdermins (hereafter abbreviated GSDMD^1–275^ and GSDME^1–270^) dramatically suppressed yeast growth rates (Fig. 1a) and colony-forming abilities (Fig. 1b) of transformants, and led to permeabilization of their plasma membranes (Fig. 1c). This observation was consistent with the findings from experiments in mammalian cells showing that the N-terminal portions of gasdermins were the active domains responsible for inducing pyroptosis^10,42^. Although western blot analysis indicated successful expression of GSDME and GSDME^1–270^, GSDMD and GSDMD^1–275^ could barely be detected (Fig. 1d). To explore whether this reflected weak expression of GSDMD and its truncation mutant, or low affinity of the monoclonal antibody (which was generated using full length protein as the immunogen), we fused a FLAG tag to either side of full-length and N-terminal fragments of gasdermins. Addition of a FLAG tag to the N-terminus of GSDMD (^FLAG^GSDMD), but not its C terminus, enabled its robust detection by both anti-FLAG and anti-GSDMD antibodies, suggesting the addition of the tag to the N-terminus boosted production or stability of the RNA and/or protein in yeast and confirming the utility of the anti-GSDMD antibody. N-terminally-tagged GSDMD^1–275^ (^FLAG^GSDMD^1–275^) was also detected, although at a much lower level than the full length version, using anti-FLAG immunoblotting. C-terminal tagging of this truncation mutant did not facilitate its detection. Both tagged versions of GSDMD^1–275^ were highly active, so we infer that they were expressed. Our difficulty detecting these toxic proteins via immunoblotting matched observations of researchers who used transient transfection to express GSDMD-N proteins in mammalian cells. 293T cells that were transiently transfected with GSDMD-N expression constructs died^30,43^. Very little (or none) of the protein was detected by western blotting of cell lysates but it was detected in the media^44^, implying that GSDMD-N can be secreted from mammalian cells. If GSDMD^1–275^ was secreted into the media from yeast cells expressing it, we speculate that this may explain to our inability to detect untagged or C-terminally tagged GSDMD^1–275^ in yeast lysates and the difference in expression levels of full length versus truncated N-terminally tagged GSDMD proteins.

Adding FLAG tags to either end of intact GSDME (^FLAG^GSDME or GSDME^FLAG^) or GSDME^1–270^ (^FLAG^GSDME^1–270^ or GSDME^1–270/FLAG^) very slightly boosted their steady-state expression (Fig. 1d). To investigate the impacts of the FLAG tag fusion on the function of wild type proteins, we compared the impact on yeast viability of FLAG-tagged versus untagged gasdermins. FLAG tags on either side of full-length GSDMD and GSDME had no impact on the growth and survival of yeast (Fig. 1e–g). FLAG tags on either side of GSDMD^1–275^ further suppressed the maximal growth rate of yeast, relative to that of yeast expressing the untagged truncated protein and greatly increased PI-permeable populations (Fig. 1e,f). Transformants expressing tagged GSDMD^1–275^ proteins exhibited lower average clonogenic potential than those expressing the untagged protein, but this difference was not statistically significant (Fig. 1g). Fusion of a FLAG tag to the C-terminus of GSDME^1–270^ maintained its lethality to yeast, but the N-terminally FLAG-tagged counterpart was tolerated by yeast: adding the tag to the N-terminus of this truncation fragment completely restored the membrane integrity of yeast transformants, their clonogenic ability and the growth rates (Fig. 1e–g).

The ability of the N-terminal gasdermin fragments to compromise yeast viability suggested that pyroptotic programmed cell death could be reconstituted in yeast by cleaving full-length gasdermins to produce their lytic N-terminal portions. Since ^FLAG^GSDMD and ^FLAG^GSDMD^1–275^, and GSDME^FLAG^ and GSDME^1–270/FLAG^ retained similar or greater activities to the untagged proteins and could be detected using anti-FLAG immunoblotting, the following experiments were performed using these FLAG-tagged proteins, unless otherwise noted.

### Reconstitution of the gasdermin D-mediated pyroptotic lethality in yeast

**Figure 2:**
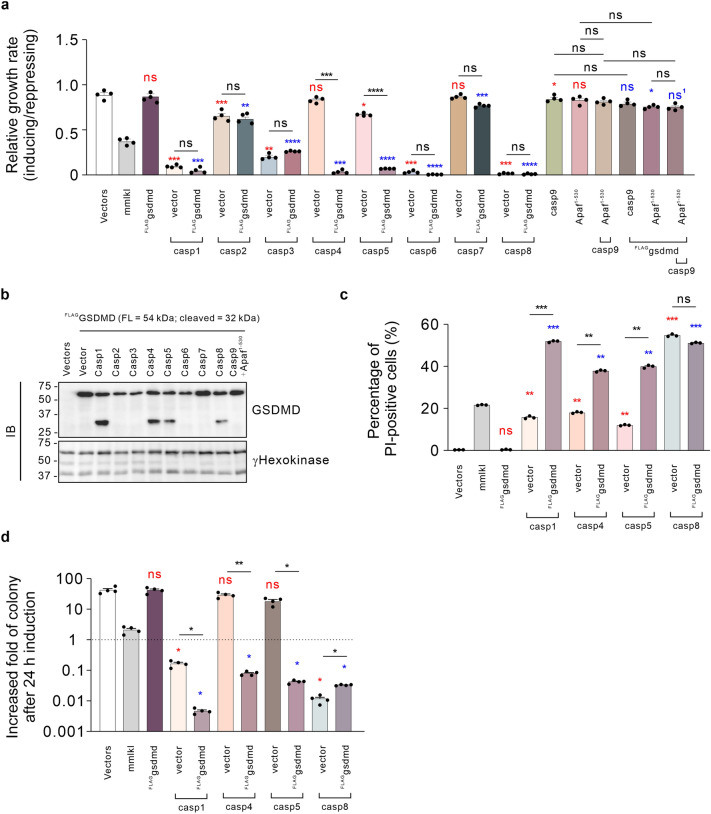
Reconstitution of GSDMD-mediated pyroptotic cell death in S. cerevisiae**. (a)** Maximal growth rates of yeast bearing expression plasmids encoding ^FLAG^GSDMD and active caspases-1 to -9, or bearing empty vectors lacking cell death genes (labeled “Vectors”), were analyzed by monitoring rate of changes in absorbance of yeast cultured in inducing and repressing liquid media. (**b)** Expression of recombinant expressed ^FLAG^GSDMD were assessed by western blot. Loading control: anti-γhexokinase. Uncropped blots are presented in Supplementary Fig. 3. (**c)** Yeast bearing expression plasmids encoding ^FLAG^GSDMD and active caspase-1, -4, -5, or -8 were grown under inducing conditions for 24 h then clonogenicity and membrane integrity were assessed. Following galactose removal, plasma membrane integrity of each yeast culture was assessed by propidium iodide (PI) uptake. (**d)** The colony-forming abilities of post-induction cultures were expressed relative to the corresponding uninduced cultures. Cells were grown in inducing media for 24 h. Following galactose removal, the post-induction cultures were diluted and plated on solid repressing media and incubated for 3 days. Data present mean ± SEM of three **c** or four independent assays (**a,d**). Differences in maximal growth rates (**a),** PI-positive population (**c)** or fold-increases in colonies (**d)** of yeast expressing empty vectors versus those expressing each transgene or sets of 2 transgenes versus individual transgenes or pairs of transgenes were compared using ANOVAs with Sidak corrections. Comparisons made between yeast expressing each transgene(s) and empty vector yeast are labeled in red; Comparisons made between each transgene(s) to yeast expressing ^FLAG^GSDMD are labeled in blue, except for ns^1^ in panel (**a)**, in which the comparator was yeast co-expressing ^FLAG^GSDMD and caspase-9. Other comparisons are indicated using black lines. *, *P* < 0.05; **, *P* < 0.01; ***, *P* < 0.001; ns, not significant.

Many caspases were found to auto-activate via overexpression in yeast^32^. To reconstitute GSDMD-mediated cell death in yeast, we initially analyzed the growth rate of yeast transformants co-expressing various human caspases with ^FLAG^GSDMD. Overexpression of pro-caspases-1, -2, -5, -8 or -9 inhibited the growth of yeast to varying extents, as did active forms of caspase-3 (fused to β-galactosidase)^45^ or caspase-6 (rearranged so its small subunit occupied the N-terminus of the protein)^46,47^ (Fig. 1a, Supplementary Fig. 2). Wild type caspase-4, and an active form of caspase-7 (missing its N-terminal 53 amino acids)^48,49^ were confirmed to be expressed in yeast (Supplementary Figs. 2, 5) but did not significantly alter yeast growth when expressed without gasdermins. Caspase-9 had a statistically significant but very subtle effect on yeast proliferation, however this marginal impact was not observed when caspase-9 was co-expressed with a constitutively active form of its adaptor, Apaf-1, composed of residues 1–530^50,51^. While previous studies revealed alterations of multi-organelle morphology^37,52^, the exact mechanism by which active caspases compromise the viability of yeast transformants remains unclear. Probably, active caspases cleave yeast substrates, which are essential to yeast physiological processes. While expression of ^FLAG^GSDMD, caspase-4, or -5 induced negligible growth inhibition, co-expression of ^FLAG^GSDMD with either caspase-4 or -5 significantly suppressed the yeast growth rate (Fig. 2a). Western blot analysis indicated successful expression of full-length ^FLAG^GSDMD and appearance of a ~ 30 kDa fragment in yeast co-expressing caspase-4 or -5 (Fig. 2b). The correlation between the expression of this cleavage product and suppressed growth rates suggests that both caspase-4 and -5 cleaved GSDMD to kill yeast, which is consistent with findings from mammalian cells documenting that caspases-4 and -5 can activate GSDMD via proteolysis^10^. It is theoretically possible that these caspases indirectly provoked the cleavage and activation of GSDMD, for example by activating a yeast protease that cleaved GSDMD, but this seems unlikely. Although co-expression of ^FLAG^GSDMD with caspase-1 or -8 did not significantly exacerbate the near-complete growth inhibition of yeast expressing these caspases alone, the ~ 30 kDa fragment of GSDMD was detected in lysates from these co-transformants (Fig. 2a,b). Co-expression of other human caspases with ^FLAG^GSDMD had no significant impact on yeast growth relative to expression of the caspases alone, and no cleavage was observed (Fig. 2a,b). These findings demonstrate a functional interaction between GSDMD and caspase-1, -4, -5, or -8 in yeast.

The inhibited growth rates of yeast co-expressing human caspases with gasdermins indicated a reduction in net proliferation, which could be a consequence of cell death, cell cycle arrest or a combination of both. To characterize the growth inhibition induced by ^FLAG^GSDMD co-expressed with either caspase-4 or -5, and to investigate the impact of ^FLAG^GSDMD cleavage by caspase-1 or -8, the membrane integrity and clonogenic ability of yeast transformants were analyzed after 24-h induction. In addition to the growth inhibition, ^FLAG^GSDMD significantly compromised the membrane integrity and colony-forming ability of yeast co-expressing caspase-4 or -5 (Fig. 2c,d). These observations are consistent with the non-canonical pyroptosis pathway in mammalian cells, in which active human caspase-4 or -5 or their murine ortholog caspase-11 proteolytically activates GSDMD^10,44,53^.

^FLAG^GSDMD greatly reduced the clonogenic abilities of yeast co-expressing caspase-1, and disrupted their plasma membranes (Fig. 2c,d). These findings complement the presence of the ~ 30 kDa fragment of GSDMD in yeast co-expressing caspase-1, implying that caspase-1 activated the pore-forming activity of GSDMD via cleavage in the yeast context, recapitulating canonical pyroptosis in mammalian cells^10^ and confirmed a recent documentation of this cleavage event in yeast^37^. Counter-intuitively, we observed that ^FLAG^GSDMD slightly improved the colony-forming ability of yeast co-expressing caspase-8, and there was a (non-significant) decrease in the proportion of PI-positive cells from these co-transformants relative to yeast only expressing caspase-8 (Fig. 2a,c,d). These discordant data hint that the intermolecular mechanism between GSDMD and caspase-8 is more complex than its interactions with caspase-1, 4 and 5, which seem to be purely cooperative in yeast.

### Reconstitution of the gasdermin E-mediated pyroptotic lethality in yeast

**Figure 3:**
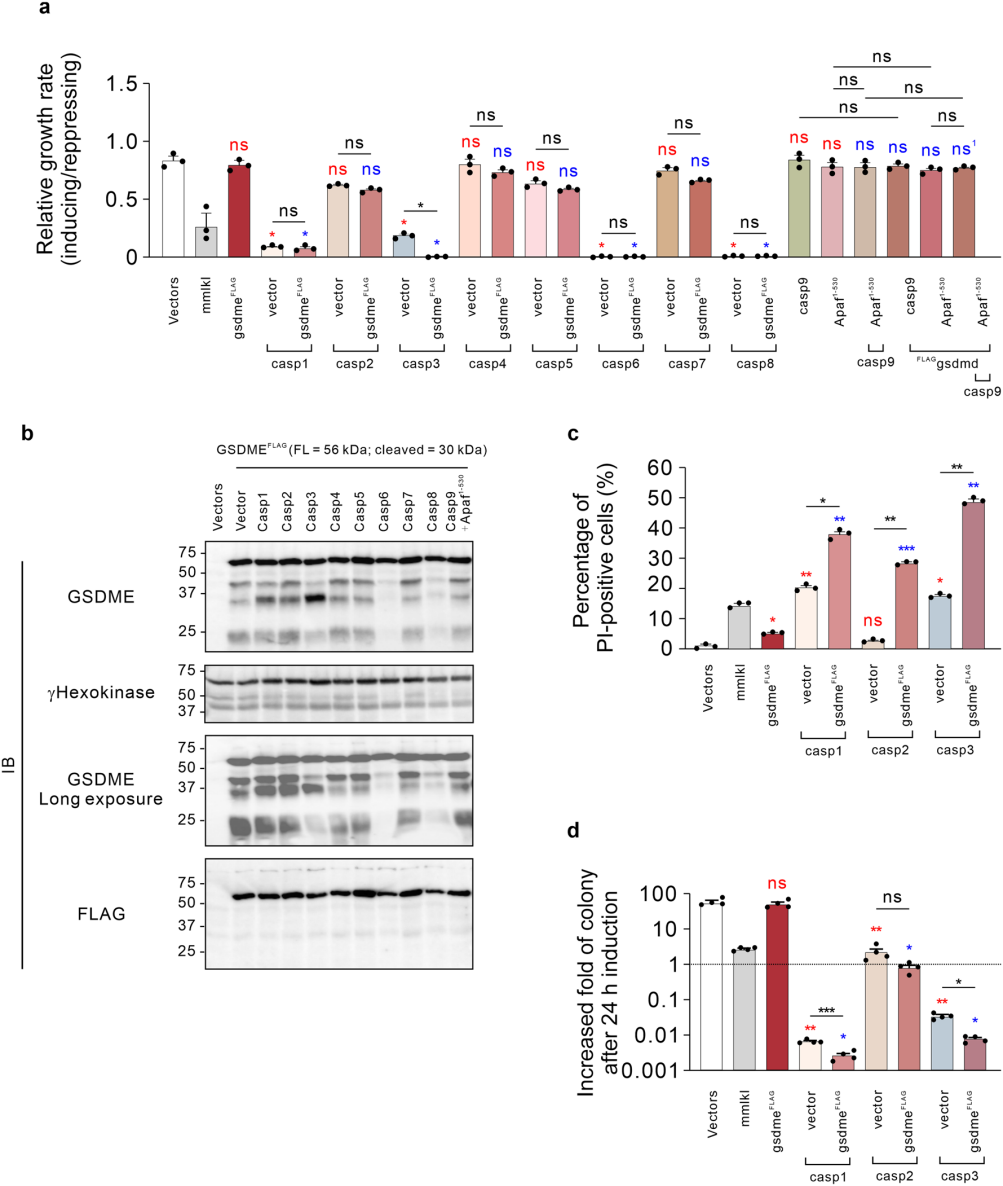
Reconstitution of GSDME-mediated pyroptotic cell death in *S. cerevisiae***. (a)** Maximal growth rates of yeast bearing expression plasmids encoding GSDME^FLAG^ and active caspases-1 to -9, or bearing empty vectors lacking cell death genes (labeled “Vectors”), were analyzed by monitoring rate of changes in absorbance of yeast cultured in inducing and repressing liquid media. (**b)** Expression of recombinant expressed GSDME^FLAG^ was assessed by western blot. Loading control: anti-γhexokinase. Uncropped blots are presented in Supplementary Fig. 4. (**c)** Yeast bearing expression plasmids encoding GSDME^FLAG^ and active caspase-1, -2, or -3 were grown under inducing conditions for 24 h then clonogenicity and membrane integrity were assessed. Following galactose removal, plasma membrane integrity of each yeast culture was assessed by propidium iodide (PI) uptake. (**d)** The colony-forming abilities of post-induction cultures were expressed relative to the corresponding uninduced cultures. Cells were grown in inducing media for 24 h. Following galactose removal, the post-induction cultures were diluted and plated on solid repressing media and incubated for 3 days. Data present mean ± SEM of three (**a,c**) or four independent assays (**d**). Differences in maximal growth rates (**a**), PI-positive population (**c**) or fold-increases in colonies (**d**) of yeast bearing empty vector versus those expressing each transgene, or sets of 2 transgenes versus individual transgenes or pairs of transgenes were compared using ANOVAs with Sidak corrections. Comparisons made between yeast expressing each transgene(s) and empty vector yeast are labeled in red; Comparisons made between each transgene(s) to yeast expressing GSDME^FLAG^ are labeled in blue, except for ns^1^ in panel (**a)**, in which the comparator was yeast co-expressing ^FLAG^GSDMD and caspase-9. Other comparisons are indicated using black lines. *, *P* < 0.05; **, *P* < 0.01; ***, *P* < 0.001; ns, not significant.

To extend the application of this yeast model to GSDME-mediated pyroptosis, we firstly analyzed the abilities of various human caspases to cooperate with GSDME by measuring yeast growth rates of single and double transformants. Fusion of the FLAG tag to the N-terminus of GSDME^1–270^ abolished its killing activity (Fig. 1d–g). Prior work demonstrated that mutation of the phenylalanine adjacent to the initiating methionine of GSDME dramatically reduced the toxicity associated with the 1–270 portion of GSDME^30^, illustrating the criticality of the GSDME N-terminus. The addition of the FLAG tag to this end of the molecule seemingly had a similar deleterious effect on its function in yeast. C-terminal FLAG-tagged GSDME was hence used to conduct subsequent experiments. While mere expression of active caspase-3 considerably suppressed the growth rate of yeast transformants, co-expression of GSDME^FLAG^ with caspase-3 substantially exacerbated the growth inhibition, suggesting that caspase-3 activated GSDME in yeast (Fig. 3a). No other human caspases showed signs of GSDME cooperation, but it is possible that the strong yeast lethality induced by expression of caspase-1, -6 or -8 limited the dynamic range of this assay (Fig. 3a). We considered evaluating caspase-mediated GSDME activation using a C-terminally tagged form of GSDME, however we failed to detect GSDME^FLAG^ in the lysates of yeast co-expressing caspases using anti-FLAG antibody (Fig. 3b). We suspect that caspases may have removed a short C-terminal portion of the protein containing the tag. However, immunoblotting using a monoclonal antibody recognizing an N-terminal epitope of GSDME provided evidence that some caspases could cleave GSDME^FLAG^ in yeast. As shown in Fig. 1d, this antibody detected four dominant proteins in induced lysates from yeast bearing the GSDME plasmid that were absent from empty vector transformant lysates. The most intense band matched the predicted size of intact GSDME^FLAG^ (56 kDa) and the others had apparent molecular weights of around 42, 30 and 23 kDa (Fig. 3b, top panel). We suspect that these smaller species resulted from cleavage of GSDME by yeast proteases. Although protein bands of approximately 30 kDa were observed in yeast expressing GSDME^FLAG^ whether a human caspase was co-expressed or not, more intense very slightly larger ~ 30 kDa bands were detected in lysates from yeast co-expressing GSDME^FLAG^ with caspase-1, -2, or -3 (Fig. 3b, top panel). Saturated exposure of the PVDF membrane confirmed that the abundance of the ~ 30 kDa fragment was elevated in these lanes (Fig. 3b, bottom panel), suggesting caspase-1, -2, and -3 cleaved GSDME^FLAG^ in yeast. Intriguingly, protein bands of around 42 and 23 kDa were much less intense in lysates from yeast co-expressing GSDME^FLAG^ with caspase-3, -6 and -8, as were the ~ 30 kDa protein bands in yeast co-expressing GSDME^FLAG^ with caspase-6 and -8 (Fig. 3b). The mechanistic basis for this is unclear.

We also analyzed the clonogenic ability and plasma membrane integrity of yeast co-expressing GSDME^FLAG^ with caspase-1, -2 or -3 to investigate the activity of the liberated 30 kDa N-terminal fragments. Interestingly, although expression of GSDME^FLAG^ did not significantly affect the growth rate and clonogenic ability of yeast, a small population of cells became PI-positive after 24 h induction (Fig. 3a,b,d). Conceivably, the small amount of the ~ 30 kDa species that we postulate resulted from cleavage by a yeast protease (in GSDME^FLAG^ transformants lacking human caspase plasmids) compromised the membranes of a minority of yeast. GSDME^FLAG^ permeabilized the plasma membrane in more yeast co-expressing caspase-3 and significantly decreased the colony-forming ability of these co-transformants (Fig. 3c,d). Together with the suppressed growth rate and enhanced production of the 30 kDa fragment, these findings indicate that caspase-3 cooperated with GSDME by liberating a 30 kDa N-terminal active domain via cleavage in yeast, consistent with findings of GSDME activation pathway in mammalian cells^30^. Interestingly, human GSDME^FLAG^ also exacerbated the permeabilization of plasma membrane and reduced clonogenic ability of yeast co-expressing caspase-1 (Fig. 3c,d). Human GSDME^FLAG^ did not significantly reduce the clonogenic ability of yeast co-expressing caspase-2, but the membrane integrity of many of these yeast was compromised (Fig. 3c,d). These observations imply that caspase-1 or caspase-2 cooperated with GSDME, generating a p30 fragment of GSDME and compromising yeast viability.

Collectively, the correlation between the increased abundance of the 30 kDa GSDME fragment and permeabilization of the plasma membrane of yeast co-expressing GSDME^FLAG^ with caspase-1, -2 or -3 suggested that GSDME-mediated pyroptotic cell death has been reconstituted in yeast.

### Inhibition of the catalytic activity of caspases optimizes the yeast model for screening for potential inhibitors

**Figure 4:**
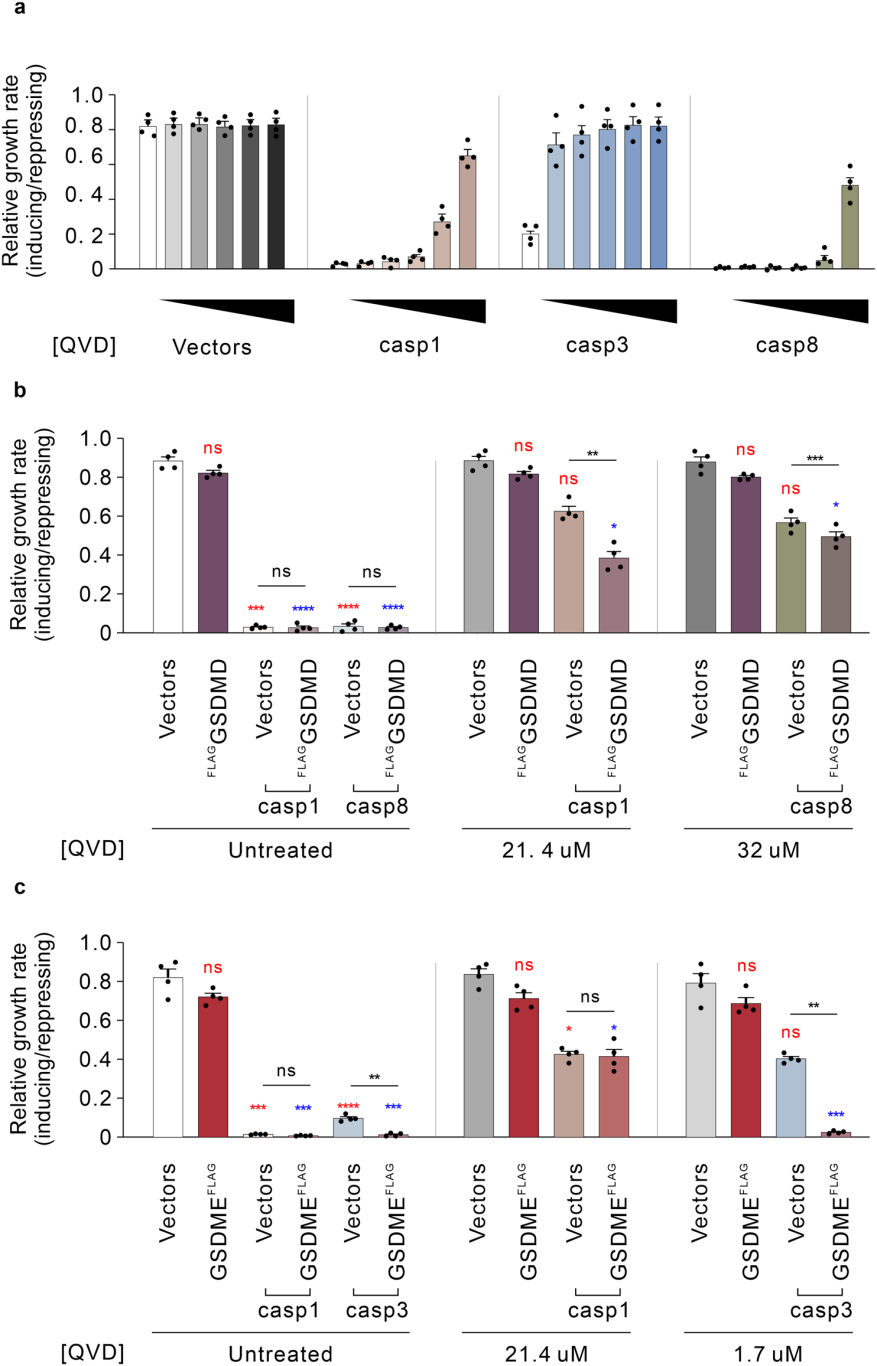
Inhibition of active caspase-induced yeast lethality expanded the applicability of the yeast expression system modeling pyroptotic cell death**. (a)** Maximal growth rates of yeast bearing expression plasmids encoding active caspases-1, -3, or -8, or bearing empty vectors lacking cell death genes (labeled “Vectors”), were analyzed by monitoring rate of changes in absorbance of yeast cultured in inducing and repressing liquid media with 0, 2, 4, 8, 16 or 32 µM of the small molecule pan-caspase inhibitor QVD. Differences in maximal growth rates of yeast bearing expression plasmids encoding (**b**) ^FLAG^GSDMD and active caspase-1, or -8; or (**c**) GSDME^FLAG^ and caspase-1, or -3, in media containing 1% DMSO with either no drug (untreated) or containing QVD at approximately half maximal inhibitory concentrations, were compared. Differences in maximal growth rates of yeast expressing empty vector versus those expressing each transgene, or sets of 2 transgenes versus individual transgenes were compared using ANOVAs with Sidak corrections. Comparisons were made between yeast exposed to the same QVD treatments. Comparisons between yeast expressing each transgene(s) and empty vector yeast are labeled in red; Comparisons between each transgene(s) to yeast expressing ^FLAG^GSDMD (**b**) or GSDME^FLAG^ (**c**) are labeled in blue. Other comparisons are indicated using black lines. *, *P* < 0.05; **, *P* < 0.01; ***, *P* < 0.001; ns, not significant.

The potent toxicity of some caspases when expressed alone limited our ability to detect any cooperation with gasdermins via further accentuation of yeast lethality. As previously reported, caspase-mediated yeast toxicity is due to protease activity^37^. Therefore, to optimize the yeast model for investigating functional caspase-gasdermin interactions, we analyzed the growth rates of yeast co-expressing gasdermins with caspase-1, -3 or -8 while partially dampening the proteolytic activity of the caspases. The pan-caspase inhibitor Q-VD-OPh (QVD) has been reported to inhibit caspase-1 to -10 in mammalian cell lines and its specificity against caspase-1 and -3 has also been tested in yeast^36,54–56^. Firstly, the dose-dependent sensitivities of caspase-1, -3 and -8 to QVD were tested using yeast grown in liquid media containing QVD at concentrations up to 32 μM. The growth rates of yeast bearing empty vectors were unaffected, suggesting that yeast tolerated QVD in liquid media up to 32 μM. While QVD potently restored the growth rate of yeast expressing caspase-3 and moderately suppressed the growth inhibition induced by caspase-1 as previously reported^36^, higher concentrations of QVD were needed to restore the growth rate of yeast expressing caspase-8. The concentrations of QVD required to restore the proliferative rates of yeast expressing caspase-1, -3 or -8 to half of that of induced, untreated yeast transformed with empty vectors (referred to below as “IC_50_”) were 21.4 μM, 1.7 μM and 30 μM, respectively (Fig. 4a).

Since our attempts to investigate the activities of co-expressed caspase-1 or -8 with ^FLAG^GSDMD were limited by the sensitivity of assays monitoring growth rates as measured by differences in absorbance over time, we tested whether minimizing caspase-induced, GSDMD-independent yeast cell death could increase the dynamic range of this assay, allowing detection of caspase-provoked, GSDMD-mediated growth inhibition. As expected, in the presence of 21.4 μM of QVD, the expression of caspase-1 only mildly, and statistically non-significantly, inhibited the growth rate, however co-expression of ^FLAG^GSDMD and caspase-1 dramatically inhibited yeast growth (Fig. 4b). The difference of maximal growth rate between yeast containing QVD-dampened caspase-1 with and without co-expressed ^FLAG^GSDMD signified caspase-1-induced GSDMD-dependent yeast lethality. This observation supported the notion that co-expression of caspase-1 with GSDMD reconstituted GSDMD-mediated pyroptotic cell death in yeast. Similarly, while the growth inhibition induced by expressing caspase-8 was suppressed by 32 μM of QVD, co-expressing ^FLAG^GSDMD exacerbated the growth inhibition achieved by QVD-ameliorated caspase-8 activity to a minor but statistically significant extent (Fig. 4b).

Intriguingly, when the growth rate of yeast expressing caspase-1 was restored to about 50% of the maximal growth rate using 21.4 μM of QVD, GSDME^FLAG^ did not further exacerbate the growth inhibition induced by caspase-1 (Fig. 4c). This was surprising given that, in the absence of QVD, GSDME^FLAG^ was cleaved by co-expressed caspase-1, and GSDME^FLAG^ expression doubled the PI-positive population and halved the colony-forming ability of yeast co-expressing caspase-1 (Fig. 3c). Perhaps the concentration of QVD used in this assay abolished the ability of caspase-1 to cleave GSDME.

Although GSDME^FLAG^ aggravated the detrimental effects on yeast of caspase-3 co-expression, the formidable caspase-3 toxicity limited the sensitivity of this yeast model to investigate protein interactions regulating caspase-3-associated pyroptosis and hampered the prospect of screening for potential inhibitors using this system. To enhance the dynamic range of our yeast model for future study of GSDME-mediated pyroptotic cell death, we analyzed the growth inhibition of yeast co-expressing caspase-3 and GSDME^FLAG^ while reducing the caspase-3-mediated yeast toxicity. Treatment using 1.7 μM (IC_50_) of QVD restored the maximal growth rate of yeast expressing caspase-3 to approximately three times that of untreated yeast, but yeast co-expressing GSDME^FLAG^ and caspase-3 while exposed to this concentration of QVD exhibited markedly reduced growth rates (Fig. 4c). This observation provided evidence that reducing caspase-3 toxicity enhanced the sensitivity of this yeast model to investigate caspase-3-induced, GSDME-mediated cell death.

Collectively, QVD-mediated reduction in the toxicity of recombinant human caspases increased the sensitivity of the model for investigating pyroptotic cell death in yeast, illustrating this system’s potential utility in screening for potential small molecule pyroptotic inhibitors.

### Evaluation of pryoptosis inhibitor drugs using yeast co-expressing caspases and gasdermins

**Figure 5:**
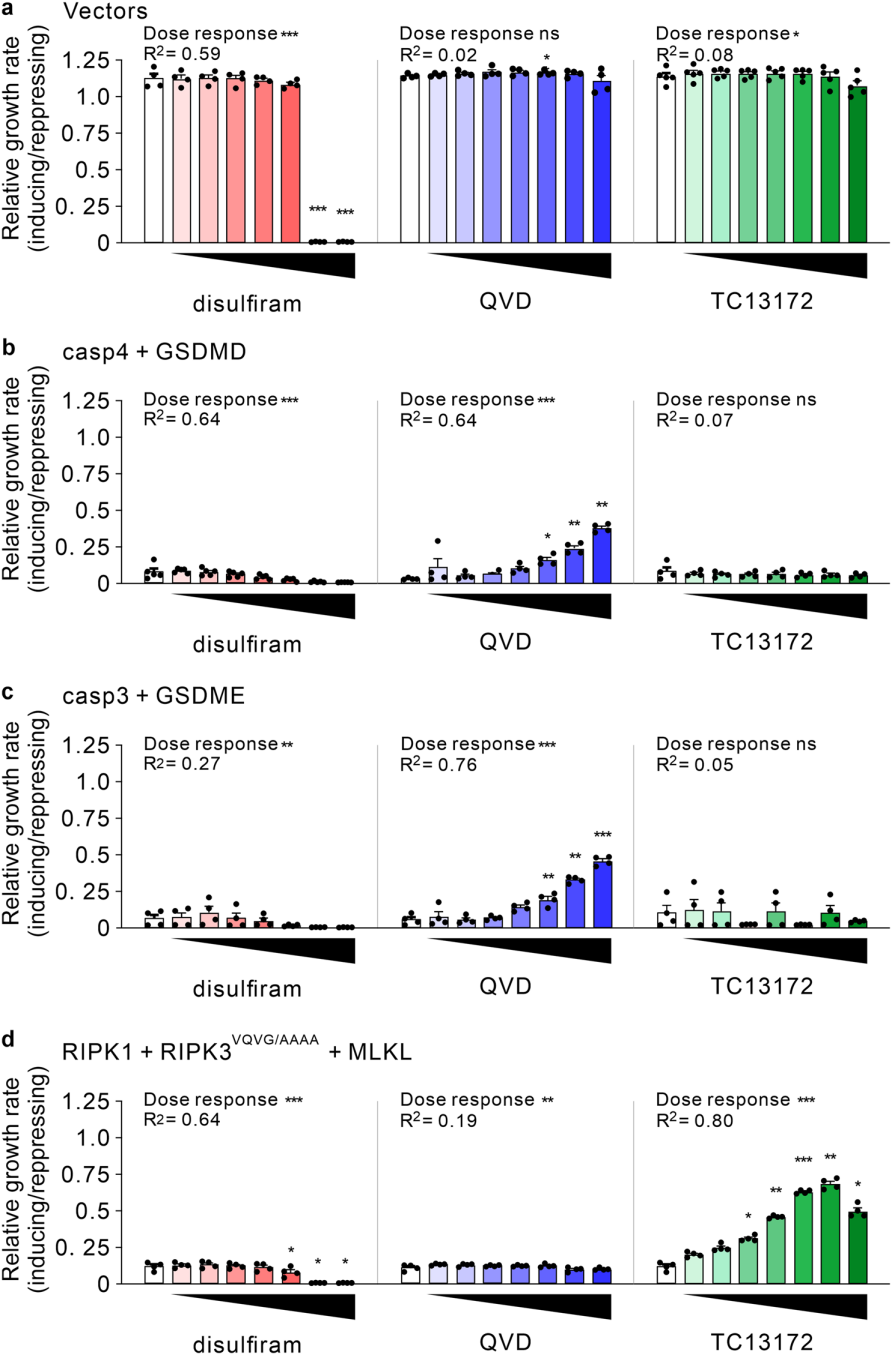
QVD enhanced the proliferation of yeast expressing caspases plus gasdermins, but tolerated concentrations of disulfiram did not. Yeast bearing empty vectors lacking cell death genes (**a**) or plasmids encoding caspase-4 plus GSDMD (**b**), caspase-3 plus GSDME (**c**) or RIPK1, a RHIM mutant of RIPK3 and MLKL (all human) (**d**) were grown in inducing or repressing liquid media containing 1% DMSO and either no drug (white columns) or 1, 2, 4, 8, 16, 32 or 64 µM of disulfiram, QVD or TC13172. The ratio of maximal growth rates in inducing versus repressing media were calculated. Data present mean ± SEM of 3–4 independent assays. Responses to each treatment were compared to untreated samples using one way ANOVAs with Sidak corrections. Statistically significant effects (*P* < 0.05) are denoted by asterisks above the columns. A test for trend was performed to identify significant drug responses (either positive or negative). R^2^ denotes the fraction of the total variance accounted for by the trend. *, *P* < 0.05; **, *P* < 0.01; ***, *P* < 0.001.

The yeast systems established in this study could conceivably be used to screen drug libraries for compounds that modulate pyroptotic signaling. To explore the feasibility of this potential application, we assessed the activity of two agents reported to target pyroptotic pathway components: disulfiram (reported to inhibit GSDMD^57^) and QVD (a caspase inhibitor^55^). The MLKL inhibitor TC13172^58^ was used as a control. QVD was well tolerated by yeast bearing empty vectors (Fig. 5a) and enhanced the proliferation of yeast expressing either caspase-4 plus GSDMD or active caspase-3 plus GSDME (Fig. 5b,c). This effect was specific, as QVD had no effect on growth inhibition caused by expression of necroptotic effectors, unlike the MLKL inhibitor TC13172, which permitted proliferation of those transformants (Fig. 5d), as was previously published^35^. Disulfiram is used to treat alcoholism^59^ and there has been recent enthusiasm for its repurposing for numerous diseases including cancer^60^, neurological^61^ and inflammatory conditions^62^. Two potential anti-inflammatory mechanisms have been proposed: suppression of gasdermin D’s pore-forming ability via modification of C^192^ of GSDMD^57^ or suppression of the upstream pyroptotic mediator NLRP3^63^. The pleiotropic activities of disulfiram probably reflect its promiscuous ability to modify cysteine residues. Indeed, many years ago disulfiram was documented to inhibit caspases-1 and -3 by covalently altering their active site cysteines^64^. Disulfiram’s tendency to provoke oxidative stress was reported to hamper the growth of the *S. cerevisiae* strain SP4^65^. We found that empty vector W303α transformants were even more sensitive (Fig. 5a). Disulfiram concentrations of up to 16 µM enabled proliferation of empty vector transformants (Fig. 5a) yet failed to counteract the growth suppression stimulated by co-expression of caspase-4 plus GSDMD (Fig. 5b). Hence, the previously reported GSDMD inhibitory property of disulfiram was not recapitulated in this yeast system.

## Discussion

Endogenous gasdermin activity is precisely regulated in vivo, as it contributes to the release of pro-inflammatory factors, whose dysregulation can cause autoinflammatory diseases, metabolic syndromes, acute and chronic inflammations^66^. In addition to regulating the release of pro-inflammatory cytokines and cellular contents, pyroptosis induces membrane rupture by forming gasdermin-assembled pore-structures on the plasma membrane^9,15,67^. We hypothesized that the ability of cytotoxic GSDMD or GSDME to permeabilize mammalian plasma membranes may heterologously extend to yeast cells. Therefore, compromised yeast viability may reflect the reconstitution of pyroptotic pathways in yeast, providing a relatively naïve biological platform to complement the current cell line-based and in vitro methods to study pyroptotic cell death.

Pyroptosis was originally identified as an immune response to pathogen infection. Recent studies reveal that pyroptosis can also be triggered via apoptosis pathways. Once activated, caspase-8 activates GSDMD, while caspase-3 activates GSDME. These cleaved gasdermins can then compromise cell membrane integrity. The activities of human caspases when exogenously expressed in yeast have been reported previously^24,32,52^. Here, we present reconstitution of pyroptosis in yeast, utilizing previously characterized active forms of caspases in a stimulus-independent manner.

As documented previously, human caspase activity can impair yeast proliferation, clonogenic potential and plasma membrane integrity^32,52^. To date, the yeast substrates whose cleavage accounts for these effects have not been identified, although a reduction in the level of full length Bni-1 after induction of caspases^37^ may have been due to direct proteolysis. We suspect that multiple yeast proteins, which are crucial for proliferation and/or plasma membrane integrity, are susceptible to proteolysis by human caspases, and that some human caspases cleave some yeast substrates more efficiently than others. This probably explains our observation that similar proportions of yeast lost membrane integrity following expression of caspases-1, -4 or -5, however caspase-1 had a much more severe impact than caspases-4 or 5 on clonogenic survival and growth rates. This finding suggests that caspase-1 may impinge on yeast proliferation and survival through cleavage of distinct proteins, whereas caspases-4 and -5 predominantly target yeast proteins required for maintaining the plasma membrane, having relatively little affinity for proteins that control mitosis.

Our data support the findings from human cell lines, showing that human GSDMD was specifically cleaved by caspase-1, -4, -5 or -8, liberating the cytotoxic N-terminal domain (~ 30 kDa fragment) from the C-terminal auto-inhibitory domain^9,10,24^. Intriguingly, while caspase-1, -4 and -5 cleavage of GSDMD inhibited growth, induced membrane permeabilization and reduced colony-forming abilities, the functional interaction between GSDMD and caspase-8 in yeast was more complex. Lysates of yeast co-expressing caspase-8 and GSDMD contained less full length GSDMD than lysates of yeast just expressing GSDMD. The ~ 30 kDa cleavage product was detectable in these lysates, but its intensity was relatively low, implying that (as in mammalian cells^24^) caspase-8 can produce the toxic N terminal fragment of GSDMD but can also cleave within this fragment, incapacitating it. Co-expression of GSDMD with caspase-8 weakly suppressed the yeast growth rate (detected under conditions of QVD-dampening of caspase-8-mediated lethality), but slightly increased the colony-forming ability, and showed a statistically non-significant trend of protection with respect to membrane integrity. Further work will be needed to determine the mechanistic basis for this apparent antagonism of caspase-8’s lethality by co-expression of GSDMD, despite these proteins cooperating to slow proliferation. It is conceivable that substrate competition may contribute to the antagonism: perhaps caspase-8-mediated cleavage of essential yeast substrates is less efficient in the presence of the alternative substrate GSDMD, and the ability of caspase-8 to cleave within the toxic N-terminal portion as well as between the amino and carboxyl domains^24^ mitigates the lethality associated with co-expression.

The correlation between caspase-3 expression and detection of a 30 kDa fragment mirrored the functional interaction between caspase-3 and GSDME in human cells: activated caspase-3 specifically cleaved GSDME, indicating the successful reconstitution of GSDME-mediated pyroptotic cell death in yeast. Interestingly, we also observed GSDME cleavage by caspase-1 or -2, which has not previously been reported in mammalian cells, although this seemed less efficient than GSDMD cleavage by caspase-1, or GSDME cleavage by caspase-3. Membrane permeabilization in yeast co-expressing GSDME with caspase-1 or -2 suggests that, in consistent with caspase-3 cleavage of GSDME, both caspase-1 and -2 could proteolyse GSDME to liberate the cytotoxic fragment in yeast. The observation that GSDME compromised the clonogenic ability of yeast co-expressing caspase-1 further supported the notion that caspase-1 activates GSDME, at least in a relative naïve eukaryotic environment. Co-expression of caspase-1 or -2 and GSDME in yeast significantly exacerbated the loss of membrane integrity, consistent with caspase-cleaved GSDME further weakening membranes that were already compromised due to direct caspase action on particular yeast substrates that play roles in maintaining membrane integrity. However, these caspases did not significantly cooperate with GSDME to reduce proliferative rates, presumably because cleaved GSDME did not directly affect the cell cycle, and maximum growth rates (early during the culture period) were not significantly affected by a minority of cells ultimately succumbing to a lytic death. Reflecting the interplay between proliferation and death, co-expression of GSDME slightly diminished the clonogenic potential of yeast expressing these caspases. This was statistically significant in the case of caspase-1 but not for caspase-2.

In addition to the undefined 45 kDa and 23 kDa GSDME fragments, we observed that expression of GSDME without caspases in yeast generated a small amount a ~ 30 kDa fragment and a weak membrane-permeabilizing activity, implying that an unknown mechanism may exist in yeast which processes GSDME in the absence of human caspases. This is supported by previous findings that caspase-3-like activities exist in *S. cerevisiae*, as reviewed by Wilkinson, D and Ramsdale, M^68^. When GSDME was co-expressed with caspase-6 or -8, we observed reduced abundance of the 45, 30 and 23 kDa proteins. Co-expression of GSDME with caspase-3 also reduced the intensity of the 45 and 23 kDa GSDME bands, while boosting the abundance of the ~ 30 kDa fragment. Due to the intense yeast killing activities of caspase-3, -6 or -8, it is possible that overexpression of the lethal caspases abrogated the biological activities of endogenous yeast proteases, including any that could cleave GSDME. Alternatively, the reduction in signals corresponding to these fragments may simply reflect additional proteolysis; perhaps caspases-3, -6 and -8 can access sites near the N-terminus of the 45, 30 and 23 kDa fragments of GSDME, where the antibody’s epitope is located.

Collectively, caspase-3 activated GSDME via cleavage in yeast, modeling GSDME-mediated pyroptosis in human cell lines. The findings that caspase-1 or -2 induced cleavage of GSDME in yeast suggest that these proteases may be capable of activating GSDME-mediated pyroptosis in mammalian cells where they are co-expressed, under circumstances where those caspases are activated.

Although the functional link between caspase expression and ~ 30 kDa fragments of GSDMD or GSDME mirrored gasdermin activation in human cells, where activated pyroptotic caspases bind to and cleave gasdermins, the detrimental effects of the lethal caspases complicated and limited our attempts to model gasdermin-mediated cell death in yeast. Dampening the toxicity of caspases was shown to improve the sensitivity of absorbance-based assays to detect cooperation between caspases and gasdermins.

A previous report identified the inhibitory effects of pan-caspase inhibitor, Q-VD-OPh (QVD), to the heterologously expressed human caspase-1 and -3 in yeast^36^. Here, we demonstrated that QVD also inhibited caspase-8-mediated yeast toxicity in a dose-dependent manner. Our results showed that partially inhibiting the proteolytic activities of caspase-1, -3 or -8 by QVD reduced caspase-mediated yeast lethality, which facilitated investigation of gasdermin-dependent cell death mediated by highly lethal caspases. This approach could be used to optimize the yeast model investigating protein interactions and screening for potential inhibitors, by maximizing the dynamic ranges of yeast growth rates.

We hope that *S. cerevisiae* bearing reconstituted pyroptotic pathways may help researchers to identify and characterize mutations, proteins or compounds that influence these pathways. The utility of this approach for screening drug libraries to identify potential pyroptotic inhibitors was explored by testing the ability of two published inhibitors to counteract the proliferative defect triggered by caspase-mediated activation of GSDMD or GSDME. As expected, the caspase inhibitor QVD enhanced the growth of yeast co-expressing caspases-4 or -3 with GSDMD or E. However, this action was inefficient: even 64 µM of QVD only exerted a partial effect. We also attempted to confirm the reported GSDMD-inhibitory activity of disulfiram in this system. Unfortunately, as had been previously documented^65^, disulfiram was toxic to yeast lacking human cell death pathway genes. This toxicity limited disulfiram levels that could be assessed for GSDMD-inhibitory capability to concentrations up to only twice the published IC_50_ for human THP-1 cells undergoing nigericin-induced pyroptosis^57^. At these concentrations, disulfiram failed to reverse the effect of caspase-4/GSDMD co-expression on yeast proliferation. These observations reinforced an impression from prior work assembling necroptotic and apoptotic pathways in yeast: that yeast bearing mammalian cell death pathways can accurately but insensitively model the actions of drugs targeting those pathways. This study and previous work^35,69^ demonstrated that potent inhibitors of pro-survival Bcl-2 relatives, MLKL, RIPK1 and caspases exerted their expected effects on yeast reconstituted with apoptotic, necroptotic and pyroptotic pathways, however the drug concentrations required to achieve those effects were substantially higher than those required to regulate cell death in mammalian cells, and some less potent inhibitors failed to protect yeast from apoptotic, necroptotic or pyroptotic death. It therefore seems unlikely that yeast reconstituted with mammalian cell death pathways would enable identification of initial low affinity “hits” from small molecule libraries. However, these systems may be useful in subsequent drug development stages, to identify derivatives with optimal activity and specificity.

In conclusion, we reconstituted GSDMD- and GSDME-mediated pyroptotic cell death in yeast, confirming hitherto identified caspase-mediated GSDMD and GSDME activation mechanisms. We also revealed the potential for GSDME activation to be mediated by caspase-1 or -2. This system provides a useful biological model to investigate mechanisms regulating pyroptosis and identify or characterize small molecules or proteins targeting specific pyroptosis components.

## Material and methods

### Plasmid construction

The following plasmids have been previously reported: pGALL-(*LEU2*)^51^, pGALS-(*LEU2*)^45^, pGALL-(*URA3*)^45^, pGALL-(*HIS3*)^51^, GALL-(LEU2)-caspase-2^49^, pGALL-(LEU2)-caspase-3–LacZ^49^, pGALL-(LEU2)-caspase-4^49^, pGALL-(LEU2)-rev-caspase-6^47^, pGALL-(LEU2)-caspase-7^53^ ^49^, pGALL-(LEU2)-caspase-9^51^, pGALL-(HIS3)-Apaf-1^1–530^^51^, pGALL-(*LEU2*)-mMLKL^35^, pGALL-(*TRP1*)-hRIPK1^35^, pGALL-(*URA3*)-hRIPK3^VQVG/AAAA^^35^, pGALL-(*LEU2*)-hMLKL^35^.

Plasmids controlled by truncated (pGALS) Gal 1/10 promoter were constructed to limit the expression of caspase-1, caspase-5 and caspase-8^70^. pGALS-(*LEU2*)-caspase-1 was constructed by inserting the BamHI/XbaI fragment from pGALL-(*LEU2*)-caspase-1^71^ into BamHI/XbaI-digested pGALS-(*LEU2*). pGALS-(*LEU2*)-caspase-5 was constructed by inserting the BamHI/XbaI fragment from pGALL-(LEU2)-caspase-5^49^ into BamHI/XbaI-digested pGALS-(*LEU2*). pGALS-(*LEU2*)-caspase-8 was constructed by inserting the BamHI/XbaI fragment from pGALL-(*HIS3*)-caspase-8^49^ into BamHI/XbaI-digested pGALS-(*LEU2*).

Plasmid bearing human GSDMD and GSDME were purchased from Addgene (Watertown, MA, USA). pET-SUMO-hGSDMD^72^ was a gift from Hongbo Luo (Addgene plasmid #111,559; http://n2t.net/addgene:111559 ; RRID:Addgene_111559). pLVX-Puro-hGSDME^73^ was a gift from Judy Lieberman (Addgene plasmid #154,876; http://n2t.net/addgene:154876; RRID:Addgene_154876). pGALL-(LEU2)-hGSDMD was made by amplifying the coding region of human *gsdmd* with primers #2117 and #2118, digesting the product with BamHI and XbaI and ligating into pGALL-(LEU2) cut with BamHI and XbaI. pGALL-(*LEU2*)-hGSDME was made by amplifying the coding region of human *gsdme* with primers #2120 and #2121, digesting the product with BamHI and XbaI and ligating into pGALL-(*LEU2*) cut with BamHI and XbaI. pGALL-(*LEU2*)-hGSDMD^1–275^ was made by amplifying pGALL-(*LEU2*)-hGSDMD with primers #2117 and #2119, digesting the product with BamHI and XbaI and ligating into pGALL-(*LEU2*) cut with BamHI and XbaI. pGALL-(*LEU2*)-hGSDME^1–270^ was made by amplifying pGALL-(*LEU2*)-hGSDME with primers #2120 and #2122, digesting the product with BamHI and XbaI and ligating into pGALL-(*LEU2*) cut with BamHI and XbaI.

pGALL-(*LEU2*) plasmids encoding either side FLAG-tagged GSDMD or GSDME were made by amplifying the target inserts with primer pairs from plasmid templates, digesting the products with BamHI and XbaI and ligating into pGALL-(*LEU2*) cut with BamHI and XbaI. The primer pairs (listed in Table 1) and templates used for amplifications of target inserts were:

**Table 1:** Primer pairs were designed to generate insert(s) from DNA template(s) via PCR.

Target plasmid	Primer sequence 5′–3′	Template	Number
pGALL-(*LEU2*)-hGSDMD	GCGGATCCGCCATGGGGTCGGCCTTTGAGCG	Human *gsdmd*	#2117
	TCGTCTAGATTAGTGGGGCTCCTGGCTCAGTC		#2118
pGALL-(*LEU2*)-hGSDME	GCGGATCCGCCATGTTTGCCAAAGCAACCAGG	Human *gsdme*	#2120
	TCGTCTAGATTATGAATGTTCTCTGCCTAAAG		#2121
pGALL-(*LEU2*)-hGSDMD^1–275^	GCGGATCCGCCATGGGGTCGGCCTTTGAGC	pGALL-(*LEU2*)-hGSDMD	#2117
	TCGTCTAGATTAATCTGTCAGGAAGTTGTGGAG		#2119
pGALL-(*LEU2*)-hGSDME^1–270^	GCGGATCCGCCATGTTTGCCAAAGCAACCAGG	pGALL-(*LEU2*)-hGSDME	#2120
	TCGTCTAGATTAATCTGGCATGTCTATGAATG		#2122
pGALL-(*URA3*)-^FLAG^hGSDMD	GCGGATCCGCCGACTACAAAGACGATGACGACAAGGGGTCGGCCTTTGAGCG	pGALL-(*LEU2*)-hGSDMD	#2156
	CTTTATTATTTTTATTTTATTGAGAGGGTGG		#1776
pGALL-(*URA3*)- hGSDMD^FLAG^	CCACTTTAACTAATACTTTCAACATTTTCGG	pGALL-(*LEU2*)-hGSDMD	#1864
	AAATCTAGATTACTTGTCGTCATCGTCTTTGTAGTCGTGGGGCTCCTGGC		#2157
pGALL-(*URA3*)-^FLAG^hGSDMD^1–275^	GCGGATCCGCCGACTACAAAGACGATGACGACAAGGGGTCGGCCTTTGAGCG	pGALL-(*LEU2*)-hGSDMD^1–275^	#2156
	CTTTATTATTTTTATTTTATTGAGAGGGTGG		#1776
pGALL-(*URA3*)-hGSDMD^1–275/FLAG^	CCACTTTAACTAATACTTTCAACATTTTCGG	pGALL-(*LEU2*)-hGSDMD^1–275^	#1864
	AAATCTAGATTACTTGTCGTCATCGTCTTTGTAGTCATCTGTCAGGAAGTTGTGGAG		#2158
pGALL-(*URA3*)-^FLAG^hGSDME	GCGGATCCGCCATGGACTACAAAGACGATGACGACAAGTTTGCCAAAGCAACCAGG	pGALL-(*LEU2*)-hGSDME	#2159
	CTTTATTATTTTTATTTTATTGAGAGGGTGG		#1776
pGALL-(*URA3*)- hGSDME^FLAG^	CCACTTTAACTAATACTTTCAACATTTTCGG	pGALL-(*LEU2*)-hGSDME	#1864
	AAATCTAGATTACTTGTCGTCATCGTCTTTGTAGTCTGAATGTTCTCTGCCTAAAGC		#2160
pGALL-(*URA3*)-^FLAG^hGSDME^1–270^	GCGGATCCGCCATGGACTACAAAGACGATGACGACAAGTTTGCCAAAGCAACCAGG	pGALL-(*LEU2*)-hGSDME^1–270^	#2159
	CTTTATTATTTTTATTTTATTGAGAGGGTGG		#1776
pGALL-(*URA3*)-hGSDME^1–270/FLAG^	CCACTTTAACTAATACTTTCAACATTTTCGG	pGALL-(*LEU2*)-hGSDME^1–270^	#1864
	AAATCTAGATTACTTGTCGTCATCGTCTTTGTAGTCATCTGGCATGTCTATGAATGC		#2161

pGALL-(*LEU2*)-^FLAG^hGSDMD: #2156 / #1776 from pGALL-(*LEU2*)-hGSDMD.

pGALL-(*LEU2*)-hGSDMD^FLAG^: #1864 / #2157 from pGALL-(*LEU2*)-hGSDMD.

pGALL-(*LEU2*)- ^FLAG^hGSDMD^1–275^: #2156 / #1776 from pGALL-(*LEU2*)-hGSDMD^1–275^.

pGALL-(*LEU2*)-hGSDMD^1–275/FLAG^: #1864 / #2158 from pGALL-(*LEU2*)-hGSDMD^1–275^.

pGALL-(*LEU2*)-^FLAG^hGSDME: #2159 / #1776 from pGALL-(*LEU2*)-hGSDME.

pGALL-(*LEU2*)-hGSDME^FLAG^: #1864 / #2160 from pGALL-(*LEU2*)-hGSDME.

pGALL-(*LEU2*)- ^FLAG^hGSDME^1–270^: #2159 / #1776 from pGALL-(*LEU2*)-hGSDME^1–270^.

pGALL-(*LEU2*)-hGSDME^1–270/FLAG^: #1864 / #2161 from pGALL-(*LEU2*)-hGSDME^1–270^.

All inserts were verified by capillary electrophoresis Sanger sequencing (Macrogen, Seoul, South Korea).

### Antibodies and reagents

Antibodies used for immunoblotting in this study were: rabbit anti-GSDMD antibody EPR19829 (Abcam, Cambridge, UK), rabbit anti-DFNA5/GSDME antibody EPR19859 (Abcam,), rabbit anti-γHexokinase #4959–9988 (Bio-Rad, Hercules, CA, USA), mouse anti-FLAG T0003 (Affinity Biosciences, Cincinnati, OH, USA), rabbit anti-caspase-1 (A-19) (Santa Cruz Biotechnology, Dallas, TX, USA), rat anti-caspase-2 #MAB3507 (Merck, Darmstadt, Germany), mouse anti-caspase-3 (clone 19) (BD BioSciences, Franklin Lakes, NJ, USA), rabbit anti-caspase-4 #4450S (Cell Signaling Technology, Danvers, MA, USA), mouse anti-caspase-5 (4F7) MBL International, Woburn, MA, USA), rabbit anti-caspase-6 (ARC0031 (Thermo Fisher Scientific, Waltham, MA USA), rabbit anti-caspase-7 (#9492) (Cell Signaling Technology), mouse anti-caspase-8 (1C12) (Cell Signaling Technology), rabbit anti-caspase-9 (#9502) (Cell Signaling Technology), rabbit anti-mouse IgG-HRP #A9044 (Sigma Aldrich, Burlington, MA, United States), donkey anti-rabbit-HRP #NA934 (Amersham, Little Chalfont, UK).

Q-VD-OPh was purchased from SMBiochemicals (Anaheim, CA, USA). Disulfiram was purchased from Selleckchem (Houston, TX, USA). TC13172 was purchased from MedChemExpress (Monmouth Junction, NJ, USA). All drugs were dissolved in dimethyl sulfoxide (DMSO) at 10 mM. The final concentration of the DMSO solvent in the assays was 1% regardless of drug concentration.

### Yeast techniques

The *Saccharomyces cerevisiae* W303α yeast strain was used in this study. Yeast culturing and transformation were performed as previously described^35^, except that the yeast colonies were inoculated overnight in minimal selective repressing liquid media prior to expansion in complete liquid media containing 2% glucose for transformation.

### Proliferation assays

Yeast proliferation assays were performed as previously described^35^. Briefly, yeast colonies were inoculated in minimal selective repressing liquid media overnight, washed twice in TE, and incubated at Abs_620_ = 0.1 in minimal selective liquid media containing 2% w/v raffinose at 30 °C for 3 h. After incubation, each yeast suspension was sub-cultured into either minimal selective repressing liquid media as uninduced control or minimal selective inducing liquid media (in the presence or absence of chemical inhibitors). All suspensions were cultured at 30 °C for 48 h and Abs_620_ was measured every 30 min. Growth rates in inducing and repressing media were calculated in rolling five hour windows. Relative growth rates were expressed as the ratios of the maximum growth rates achieved in inducing versus repressing media).

### Membrane integrity and clonogenicity assays

Yeast membrane integrity and clonogenicity assays were performed as previously described^35^. Briefly, yeast colonies were inoculated in minimal selective repressing liquid media overnight, washed twice, and resuspended in minimal selective liquid media containing raffinose at Abs_620_ = 0.1. For each yeast transformant, 200 μl aliquots were set aside as uninduced controls and other aliquots were grown in 2 ml of minimal selective inducing liquid media at 30 °C for 24 h. After incubation, both uninduced and induced yeast were washed twice in phosphate buffered saline (PBS) and resuspended in PBS containing 50 μg/ml propidium iodide at Abs_620_ = 0.1. Flow cytometry (FACSCanto™, BD Bioscience) was performed, gating on intact cells. In the meanwhile, after incubation in minimal selective inducing liquid media, serial dilutions of both uninduced and induced yeast were performed, then plated and incubated on minimal selective repressing solid media. After three days, colonies were counted and expressed relative to the colony forming units (CFU) in the uninduced cultures.

### Western blotting

Western blots were performed as previously described with minor changes^74^. Yeast transformants were grown in minimal selective repressing liquid media overnight, washed twice in TE then incubated in 1.5 ml complete inducing liquid media for 7 h. After incubation, yeast cultures were pelleted and mixed with glass beads for approximately same volume, then frozen. Yeast pellets were directly resuspended in the appropriate volume of pre-heated yeast cracking buffer, lysed by two cycles of 1 min vortexing followed by 1 min incubation at 98℃. Yeast lysates were resolved on 15% Tris–Glycine gels. After transfer onto PVDF membranes, membranes were blocked with EveryBlot blocking buffer (#12010020, Bio-Rad, Hercules, CA, USA) for 2 min and then probed with primary antibodies. The PVDF membranes were washed then incubated in HRP-conjugated secondary antibodies. After incubation, the membranes were washed prior to detection with SignalFire™ ECL Reagent (#6883, Cell Signaling, Danvers, MA, 01923 United States).

### Statistics

Differences between groups of transformants were compared using one way ANOVAs with Sidak corrections, after checking the data were normally distributed using Shapiro-Wilk tests. Statistical analyses were performed using GraphPad Prism 9.0 (San Diego, CA, USA).

## Supplementary Information

Supplementary Information.

## Data Availability

The data used to support the findings of this study are included within the article.
