# Supplementary material for: Reconstitution of human pyroptotic cell death in Saccharomyces cerevisiae
Source: Sci Rep. 2023 Feb 22;13:3095. doi: 10.1038/s41598-023-29464-5 (PMC9946934; doi:10.1038/s41598-023-29464-5)
Supplement: Supplementary file 1 — Supplementary Information. [file 41598_2023_29464_MOESM1_ESM.docx]

Reconstitution of Human Pyroptotic Cell Death in *Saccharomyces Cerevisiae*

Yanhao Ji ^1^, and Christine J. Hawkins ^1,^*

1. Department of Biochemistry and Chemistry, La Trobe Institute for Molecular Science, La Trobe University, Bundoora, VIC, Australia

***** Correspondence: c.hawkins@latrobe.edu.au

**Supplementary Figure 1**


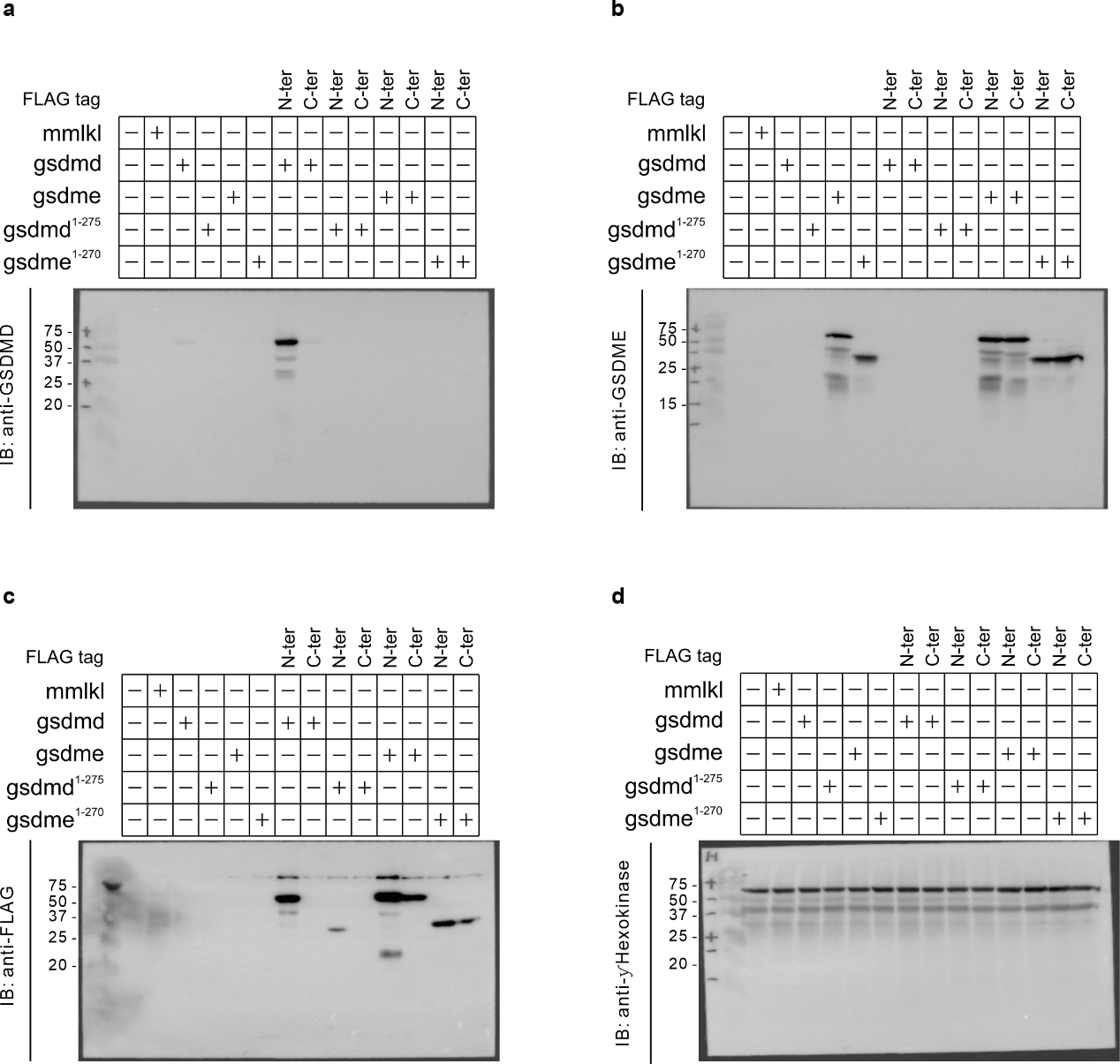


**Supplementary Figure 1**: Uncropped blots for Figure 1d. Expression of recombinant expressed wildtype or FLAG-tagged GSDMD and GSDME were assessed by western blot. Each membrane was immunoblotted by: **a** anti-GSDMD antibody; **b** anti- GSDME antibody; **c** anti-GSDMD antibody; and **d** anti-ƴHexokinase antibody. Combined white light and ECL images are shown so the edges of the blots are visible.

**Supplementary Figure 2**


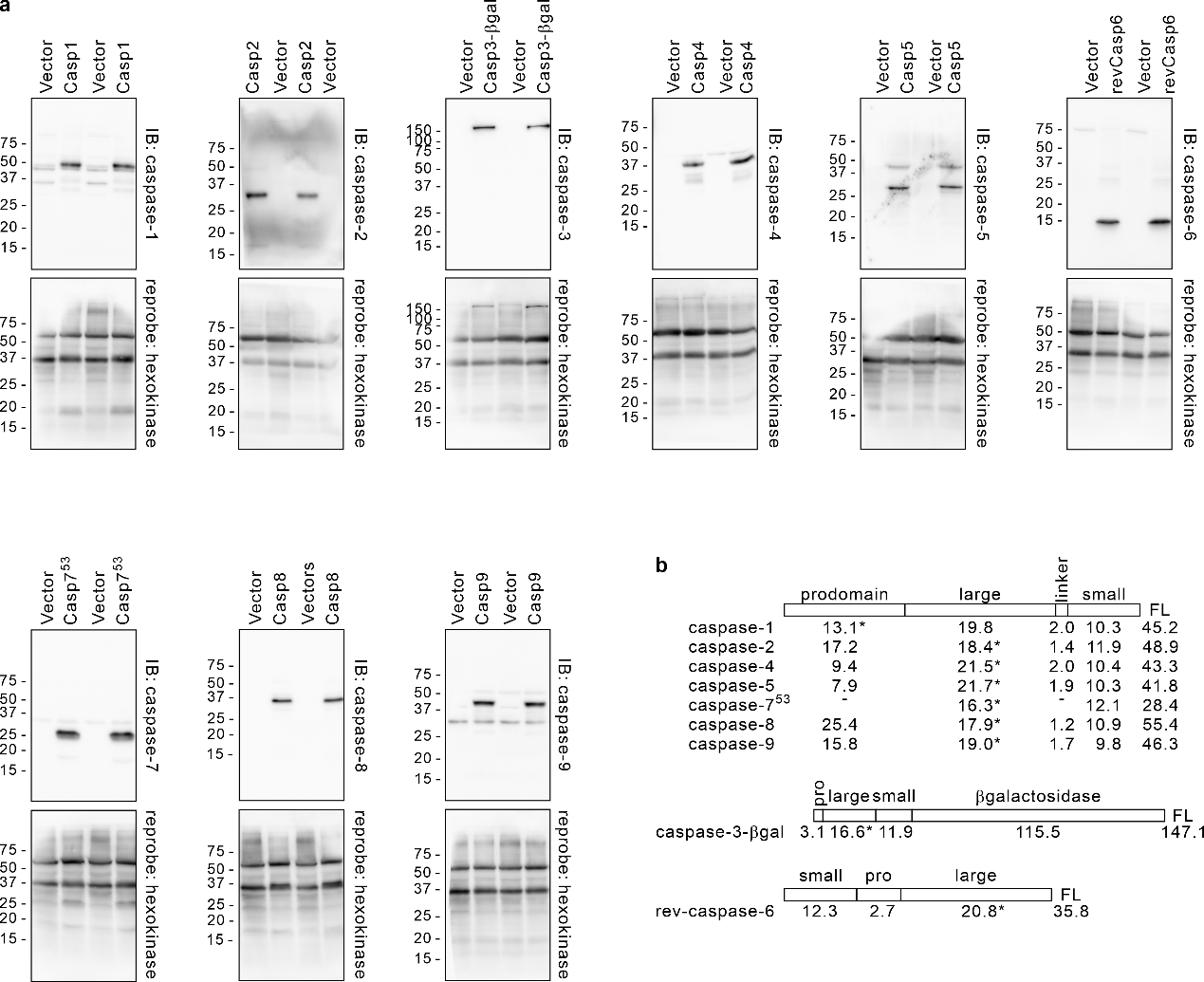


**Supplementary Figure 2**: Western blots to detect caspases in yeast. **a** Yeast were transformed with empty vector (“Vector”) or plasmids encoding active forms of human caspases-1-9. Induced lysates were subjected to SDS-PAGE then immunoblotting with antibodies recognizing each of the caspases. Blots were then re-probed with an anti-ƴHexokinase antibody. **b** Predicted molecular weights (in kDa) of the subunits of the caspases encoded by the vectors are shown; full length (“FL”) sizes are listed at the right. The domains that are recognized by the antibodies are labeled with asterisks. The residues that comprise caspase domains were obtained from the following articles: Stennicke, H., et al., J. Biol. Chem., 1999. 274(4): p. 8359-8362; Munday, N.A., et al., J. Biol. Chem, 1995. 270(26): p. 15870-15876; Taylor, R.C., S.P. Cullen, and S.J. Martin, Nat Rev Mol Cell Biol., 2008. 9(3): p. 231-41. Uncropped blots are provided in Supplementary Figure 5.

**Supplementary Figure 3**


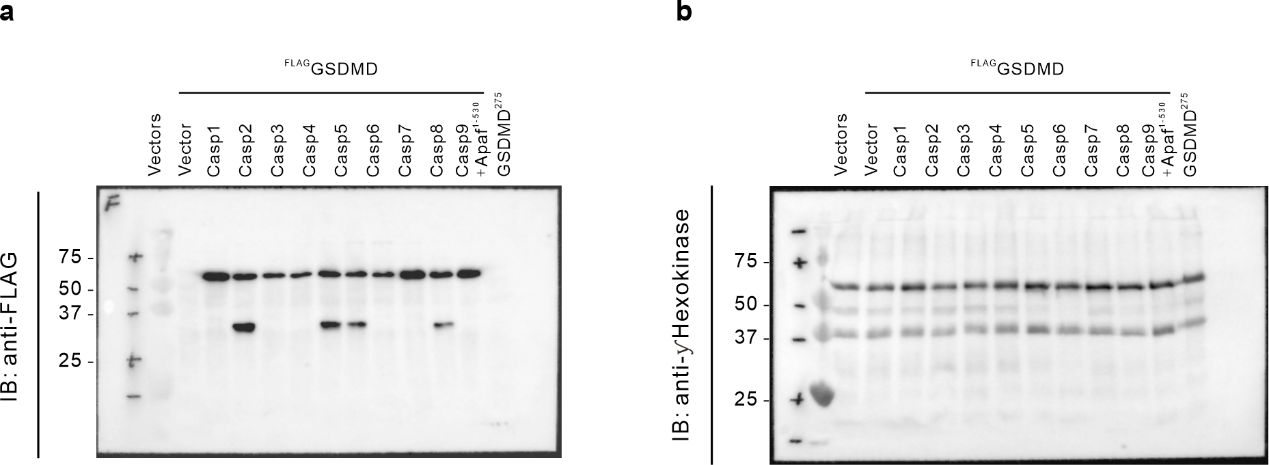


**Supplementary Figure 3**: Uncropped blots for Figure 2b. Expression of recombinant expressed ^FLAG^GSDMD were assessed by western blot. Each membrane was immunoblotted by: **a** anti-FLAG antibody; and **b** anti-ƴHexokinase antibody. Combined white light and ECL images are shown so the edges of the blots are visible.

**Supplementary Figure 4**


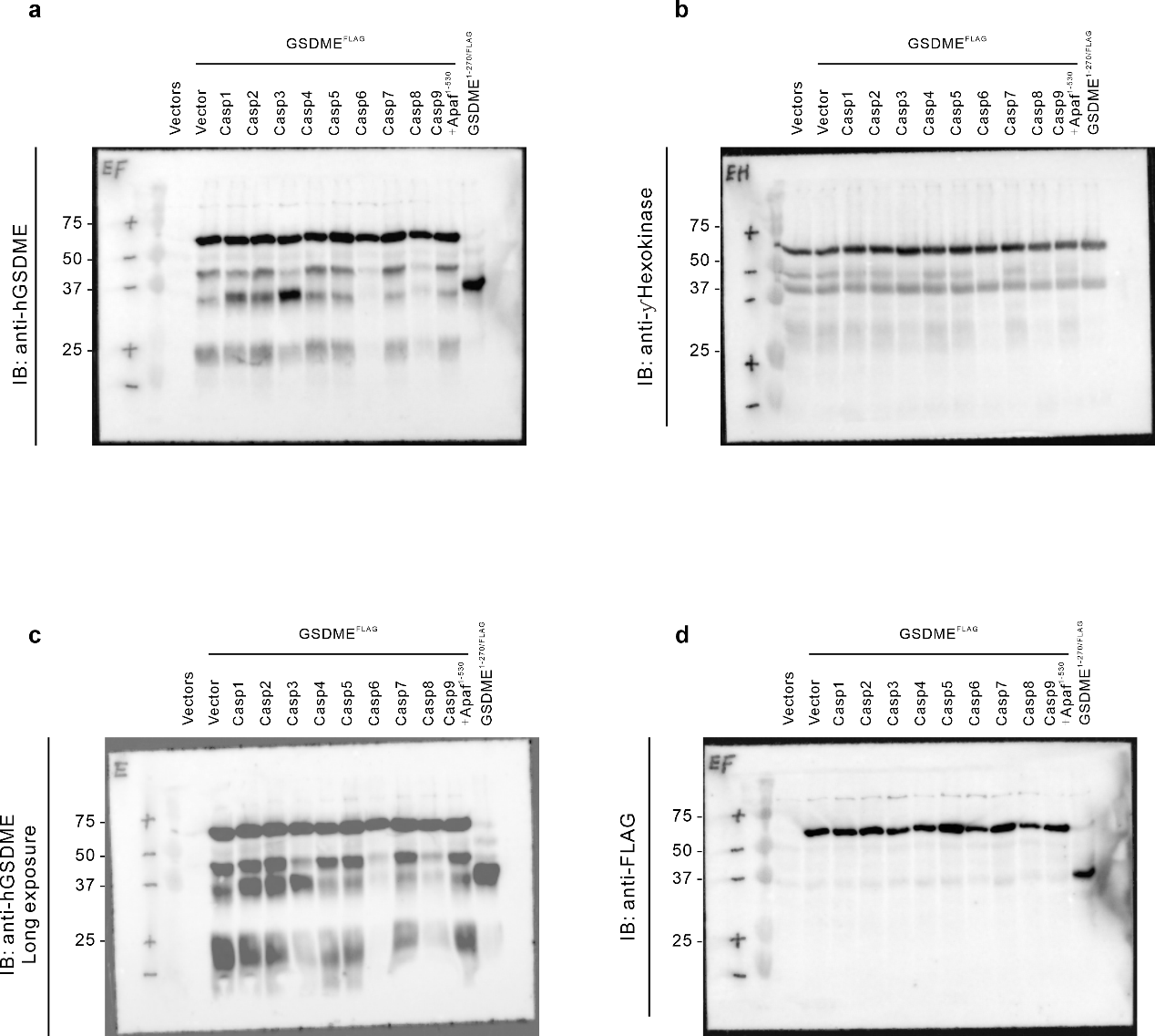


**Supplementary Figure 4**: Uncropped blots for Figure 3b. Expression of recombinant expressed GSDME^FLAG^ was assessed by western blot. Each membrane was immunoblotted by: **a** anti-hGSDME antibody; **b** anti-ƴHexokinase antibody; **c** anti-hGSDME antibody, saturated (long) exposure; and **d** anti-FLAG antibody. Combined white light and ECL images are shown so the edges of the blots are visible.

**Supplementary Figure 5**

**
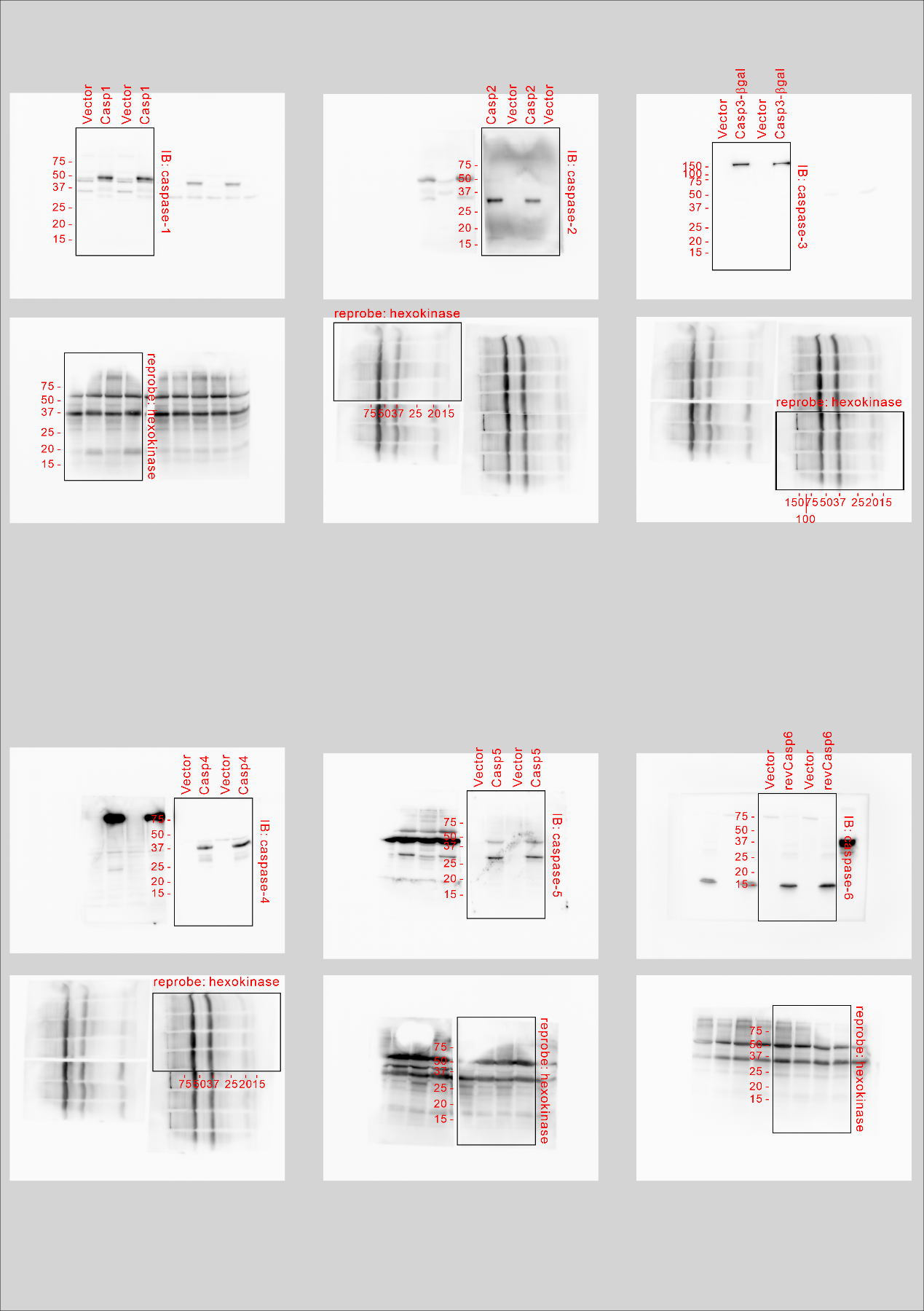
**


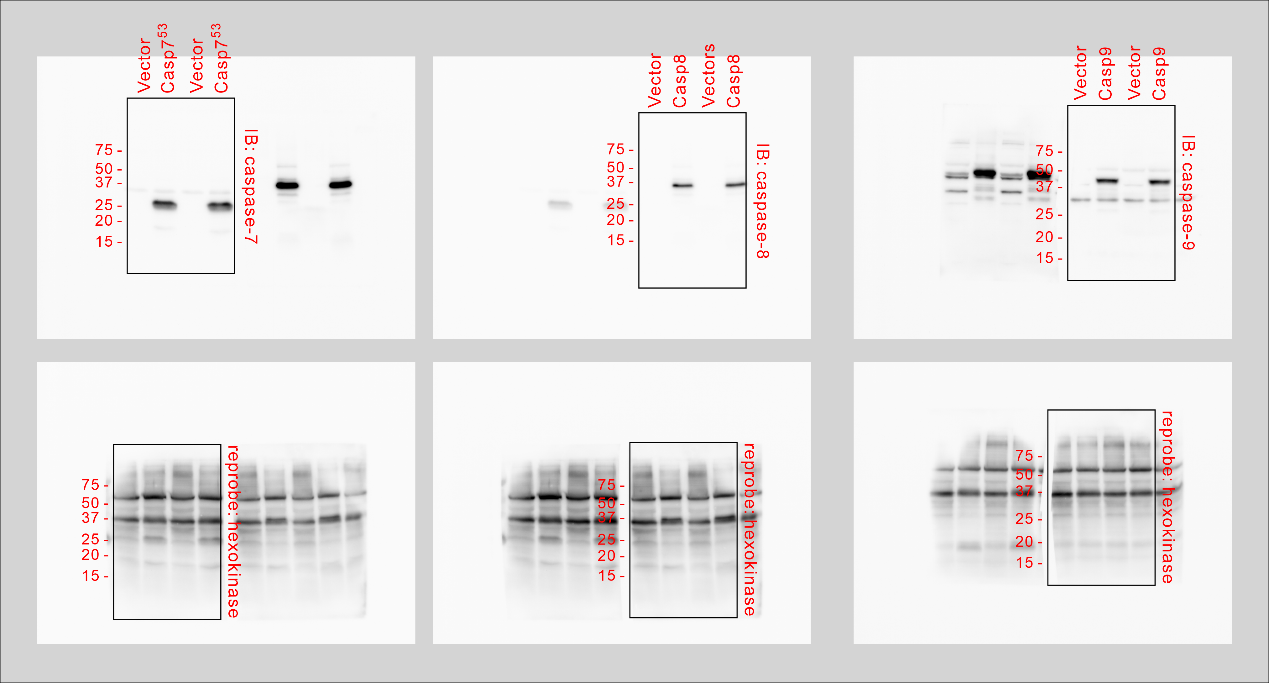


**Supplementary Figure 5**: Uncropped images used for Supplementary Figure 2.
